# Ribosome Rescue and Translation Termination at Non-Standard Stop Codons by ICT1 in Mammalian Mitochondria

**DOI:** 10.1371/journal.pgen.1004616

**Published:** 2014-09-18

**Authors:** Shiori Akabane, Takuya Ueda, Knud H. Nierhaus, Nono Takeuchi

**Affiliations:** 1 Department of Medical Genome Sciences, Graduate School of Frontier Sciences, University of Tokyo, Kashiwa-shi, Chiba, Japan; 2 Institut für Medizinische Physik und Biophysik, Charité - Universitätsmedizin Berlin, Berlin, Germany; Max Planck Institute for Biology of Ageing, Germany

## Abstract

Release factors (RFs) govern the termination phase of protein synthesis. Human mitochondria harbor four different members of the class 1 RF family: RF1Lmt/mtRF1a, RF1mt, C12orf65 and ICT1. The homolog of the essential ICT1 factor is widely distributed in bacteria and organelles and has the peculiar feature in human mitochondria to be part of the ribosome as a ribosomal protein of the large subunit. The factor has been suggested to rescue stalled ribosomes in a codon-independent manner. The mechanism of action of this factor was obscure and is addressed here. Using a homologous mitochondria system of purified components, we demonstrate that the integrated ICT1 has no rescue activity. Rather, purified ICT1 binds stoichiometrically to mitochondrial ribosomes in addition to the integrated copy and functions as a *general* rescue factor, *i.e.* it releases the polypeptide from the peptidyl tRNA from ribosomes stalled at the end or in the middle of an mRNA or even from non-programmed ribosomes. The data suggest that the unusual termination at a sense codon (AGA/G) of the oxidative-phosphorylation enzymes CO1 and ND6 is also performed by ICT1 challenging a previous model, according to which RF1Lmt/mtRF1a is responsible for the translation termination at non-standard stop codons. We also demonstrate by mutational analyses that the unique insertion sequence present in the N-terminal domain of ICT1 is essential for peptide release rather than for ribosome binding. The function of RF1mt, another member of the class1 RFs in mammalian mitochondria, was also examined and is discussed.

## Author Summary

Mammalian mitochondrial ICT1, a bacterial ArfB homolog, is interestingly an integral component of the mitoribosome (MRPL58). The mechanism of ribosome rescue by this factor was obscure and is addressed here. Utilizing a homologous mitochondria system of purified components we demonstrate that the integrated ICT1 has no rescue activity, as opposed to a previous model. Rather, purified ICT1 added to mitoribosomes has a general rescue activity; it recycles ribosomes stalled at the end or in the middle of mRNAs and can even hydrolyze peptidyl-tRNA bound to non-programmed ribosomes. These results further imply that ICT1 can function in the translation termination at non-standard stop codons AGA/G in mammalian mitochondria. Our data challenge a previous model claiming that RF1Lmt/mtRF1a is responsible for the translation termination at non-standard stop codons. A mutational study indicates that the unique insertion sequence in ICT1 is essential for peptide release. The function of RF1mt, another member of the class1 RFs in mammalian mitochondria, was also examined and is discussed.

## Introduction

Stalled ribosomes must be rescued for productive cycles of cellular protein synthesis. So far, three rescue systems for ribosomes stalled at the ends of nonstop mRNAs have been identified in bacteria. The most thoroughly characterized pathway is the *trans*-translation system involving tmRNA [1].

The second pathway engages ArfA (formerly YhdL) [2], [3], a small protein composed of approximately 55 residues. ArfA was originally identified as a factor essential for the viability of *E. coli* in the absence of tmRNA, and accordingly was renamed ArfA, for alternative ribosome-rescue factor. ArfA, in concert with RF2, takes over the rescue of stalled ribosomes, where RF2 hydrolyzes peptidyl-tRNA in a GGQ motif-dependent but codon-independent manner.

The third pathway is mediated by ArfB (formerly YaeJ) [4]. This factor is a reduced paralogue of the bacterial class 1 release factors (RFs) that retains domain 3 but lacks domains 1, 2 and 4. Domains 2 and 4 are essential for stop-codon recognition, whereas domain 3 contains the GGQ-motif. In agreement with this, the ArfB in *E. coli* hydrolyzes the peptidyl-tRNAs of ribosomes stalled at the 3′-ends of nonstop mRNAs, and functions codon-independently. The deletion of the genes of both tmRNA and ArfB is not lethal in contrast to that of tmRNA and ArfA, but the overexpression of ArfB alone rescues the double depletion of tmRNA and ArfA. Accordingly, it was suggested that this factor should be renamed ArfB, for alternative ribosome rescue factor B. The crystal structure of ArfB, bound to the *Thermus thermophilus* 70S ribosome in complex with the initiator tRNA_i_^fMet^ and a short mRNA, has been determined [5]. The N-terminal globular domain of ArfB occupies the A site of the 50S subunit next to the P-site tRNA, and its C-terminal tail occludes the mRNA tunnel downstream of the 30S A-site. The latter is thought to function as a sensor to detect a stalled ribosome, based on the occupancy of the mRNA entry channel. Indeed, ArfB prefers a ribosomal complex with a nonstop mRNA, where the A-site is vacant on the ribosome [3]. Subsequently, the binding of the tail within the mRNA entry channel allows the N-terminal globular domain to optimally position its GGQ motif in the peptidyl-transferase center (PTC) and to catalyze the hydrolysis of peptidyl-tRNA. Unlike the other class 1 RFs, ArfB contains a 25 residue insertion sequence in its N-terminal globular domain. The function of this sequence in a codon-independent peptide release factor is unknown.

We have only scarce knowledge about the ribosome rescue system in mitochondria. The so-called “immature colon carcinoma transcript-1” (ICT1) is a bacterial ArfB homolog in mammalian mitochondria; members of this factor family are widely distributed in bacteria and organelles of all eukaryotic phyla [6]. No homologs for either tmRNA or ArfA have been found in mammalian mitochondria. ICT1 catalyzes the release of formylmethionine from its P-site fMet-tRNA in *E. coli* 70S ribosomes, in a codon-independent manner [7]. ICT1 is an essential mitochondrial protein, and a mutation in the GGQ-motif of ICT1 causes loss of cell viability [7]. These observations suggested that ICT1 may be responsible for the ribosome rescue in mammalian mitochondria. Strangely, ICT1 is an integral component of the mitochondrial ribosome (mitoribosome [7], [8]). ICT1 is a member of the large mitoribosomal subunit (mitochondrial ribosomal protein L58, MRPL58), and a crucial component for its assembly [7]. No other RF family protein has been shown to be an integral ribosomal component. It is unclear, how the integrated ICT1 functions to rescue stalled ribosomes in the mammalian mitochondrial translation system.

In the present study, we have investigated the function of human mitochondrial ICT1, utilizing 55S mitoribosomes purified from pig liver mitochondria. We demonstrate that the codon-independent peptide-release activity on 55S ribosomes is only observed, when we add ICT1 to the system containing the 55S-integrated ICT1 (or to a system containing bacterial 70S ribosomes lacking integrated ICT1). These results suggest that the 55S-integrated ICT1 lacks peptide-release activity, as opposed to a previous model [7]. We further show that ICT1 can rescue ribosomal complexes not only at the ends of mRNAs, but also in the middle of mRNAs, and even without mRNAs. Our data suggest that ICT1 is a versatile ribosome rescue factor, which is also involved in the translation termination at non-standard stop codons AGG and AGA in mammalian mitochondria. The unique insertion sequence in the N-terminal domain of ICT1 is required for peptide release rather than involved in binding to the ribosome as demonstrated by a mutational analysis.

## Results

### ICT1 shows codon-independent peptide-release activity on 55S mitoribosomes, independent of the 55S-integrated ICT1

**Figure 1:**
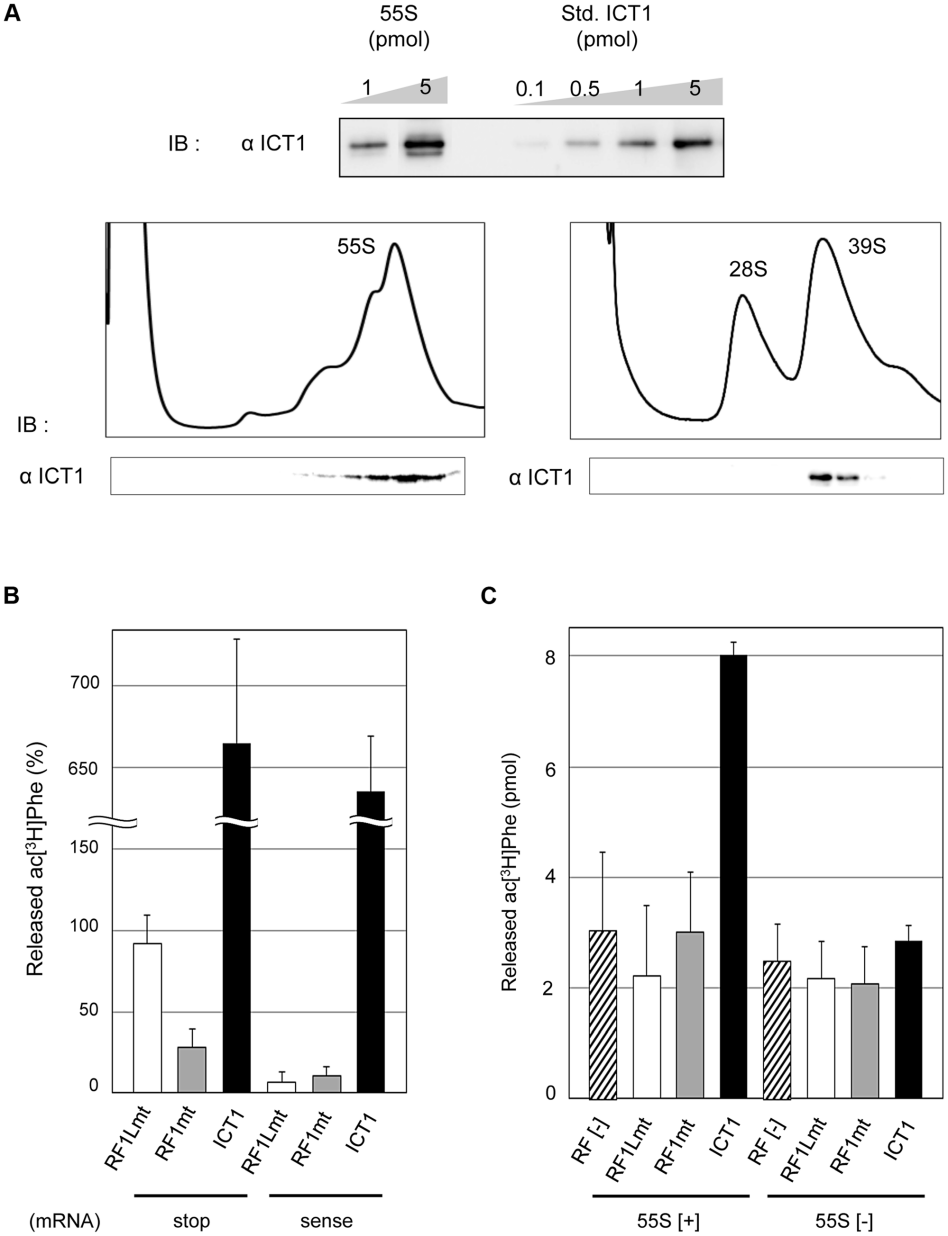
ICT1 shows codon-independent peptide-release activity on 55S mitoribosomes. (**A**) ICT1 is integrated within the 39S large mitoribosomal subunit in a 1∶1 stoichiometry. The amounts of 55S-integrated ICT1 on 55S ribosomes were estimated by Western blotting, using the purified ICT1 protein as a standard (Std. ICT1; upper panel). 55S mitoribosomes (lower left panel) or mitoribosomal subunits (lower right panel) were separated on 15%–30% (w/v) sucrose gradients (containing 10 mM Tris-HCl [pH 7.4], 80 mM NH_4_Cl, 8.2 mM MgSO_4_, and 1 mM DTT, for 55S; 10 mM Tris-HCl [pH 7.4], 200 mM KCl, 2 mM MgCl_2_, 2 mM GDP and 1 mM DTT, for subunits), and the fractions were analyzed by Western blotting with an ICT1 antibody. IB, immunoblotting. (**B**) Exogenous ICT1 exhibits codon-independent peptide-release activity on 55S mitoribosomes. The ac[^3^H]Phe release activities of ICT1, RF1Lmt and RF1mt on 55S ribosomes were tested in the presence of mRNAs and ac[^3^H]Phe-tRNA^Phe^. Ribosomes were programed by mRNAs encoding MF-UAA (stop) or MFV (sense). The results were evaluated relative to the 100% value, when all of the ac^[3^H]Phe-tRNA^Phe^ bound to ribosome was hydrolyzed; 100% value corresponds to 0.9 pmol of ac[^3^H]Phe. Results represent the average of at least three independent experiments. The error bars indicate standard deviation (SD). (**C**) ICT1 shows peptide release activity on 55S mitoribosomes in the absence of mRNA. Peptide-release assays on 55S ribosomes were performed as in (B), but in the absence of mRNA. 55S [+] and 55S [−] indicates the assays in the presence and absence of 55S mitoribosomes, respectively. Results represent the average of at least three independent experiments. The bars on the graph indicate SD.

ICT1 is a component of the 55S mitochondrial ribosome (mitochondrial ribosomal protein L58, MRPL58) [7], [8]. However, it was not clear whether the 55S ribosome itself with the integrated ICT1 exhibits the basal peptide-release activity, or whether an exogenous ICT1 can function on the 55S ribosome. To address these questions, we prepared 55S mitoribosomes from pig liver mitochondria as a model for human mitochondria, and tested them in a peptide-release assay. Pig mitochondrial ribosomes share high homology with those from human mitochondria and are easier to prepare. As shown in Fig. 1A (upper panel), 55S ribosomes contained a full complement of ICT1 in a 1∶1 stoichiometry (Fig. 1A, upper panel), which was a component of the large 39S mitoribosomal subunit (Fig. 1A, lower panel), consistent with the previous reports [7], [8].

In the peptide-release assay, a ribosomal complex was constructed using the 55S mitoribosome, mRNA encoding either MF-stop (UAA) or MFV, and N-acetyl-[^3^H]Phe-tRNA^Phe^ (ac[^3^H]Phe-tRNA^Phe^) as a model of peptidyl-tRNA. Accordingly, ac[^3^H]Phe-tRNA^Phe^ was present at the P-site, and the stop codon (UAA) or sense codon for Val (GUU) was at the A-site in the complex. The released ac[^3^H]Phe from tRNA was assessed in the presence of peptide release factors; values in the absence of release factors were taken as background and subtracted from the measured values (Fig. 1B). The mitochondrial termination factor RF1Lmt/mtRF1a showed efficient peptide-release activity with MF-stop(UAA) mRNA, but not with MFV mRNA, as expected (Fig. 1B, RF1Lmt) [9], [10]. Approximately 90% of the ac[^3^H]Phe-tRNA^Phe^ on the ribosome programmed by MF-stop(UAA) mRNA was hydrolyzed by RF1Lmt/mtRF1a. RF1mt, a class 1 RF family protein in mammalian mitochondria with an unknown function, did not show peptide-release activity for both mRNAs (Fig. 1B, RF1mt), even using an excess amount (Fig. S1). All the results with RF1mt hereafter are discussed later.

ICT1 showed the ac[^3^H]Phe release activity for both MF-stop(UAA) mRNA and MFV mRNA on 55S ribosomes (Fig. 1B, ICT1), consistent with its codon-independent peptide-release activity on 70S ribosomes [7]. Unexpectedly, the activity of ICT1 greatly exceeded the 100% value. We suspected that ICT1 performs multiple reaction cycles on the ribosome until the acPhe-tRNA input has been consumed, but perhaps with that fraction of ribosomes, which does not carry mRNA. Therefore, we performed peptide-release assays in the absence of mRNA; Fig. 1C exhibits ICT1 peptide-release activity under this condition, but only in the presence of 55S ribosomes (compare 55S[+] and 55S[−]), and even Phe is quantitatively released from Phe-tRNA when bound to non-programmed 55S ribosomes (Fig. S2A). Accordingly, most of the ICT1 activity observed in the presence of mRNA actually originated from multiple reactions on mRNA-free ribosomal complexes; ICT1 releases AcPhe in the absence of mRNA also with bacterial 70S ribosomes as does ArfB (Fig. S3). Both RF1Lmt and RF1mt are not able to release peptides from peptidyl-tRNA bound to non-programmed mitoribosomes (Fig. 1C, RF1Lmt and RF1mt). The 55S ribosomes alone (containing stoichiometric amounts of ICT1) did not show peptide-release activity in the peptide release experiments (for example, see Fig. 1C, compare RF[−] of 55S[+] and 55S[−]). These results indicated that the ICT1 integrated in the ribosome does not exhibit the peptide-release activity.

We have shown in this section that ICT1 shows codon-independent peptide release activity when added to 55S mitoribosomes; the activity is not caused by the 55S-integrated ICT1. Added ICT1 is able to exhibit peptide release activity even in the absence of mRNA. These results suggest that the 55S-integrated ICT1 is not present at the active site, where a release factor has to bind in order to trigger the release of the peptide.

### ICT1 can rescue stalled ribosomes in the middle of mRNA

**Figure 2:**
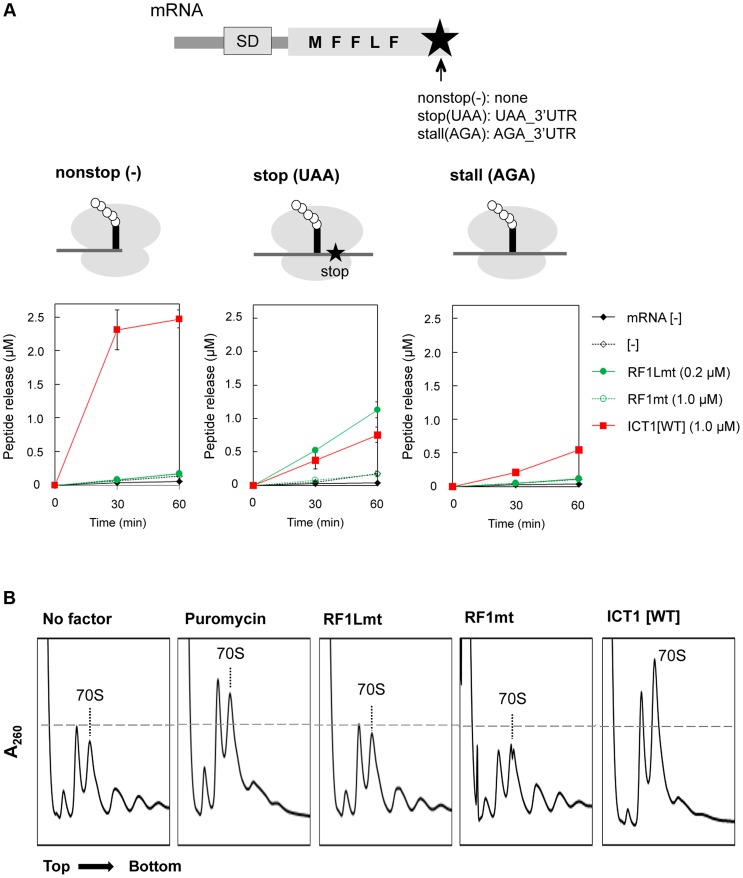
ICT1 can function in the middle of the mRNA. (**A**) ICT1 significantly functions with stop (UAA) and stall (AGA) mRNAs. Multi-round translation assays with *E. coli* 70S ribosomes were performed with the indicated peptide release factors, using mRNAs depicted in the upper. Each mRNAs differs at the 3′-terminus. Nonstop, no stop codon and no 3′UTR; stop, UAA stop codon followed by 3′UTR; sense, AGA sense codon followed by 3′UTR. The ribosomes stall at the AGA codon, since only phenylalanyl-tRNA synthetase and leucyl-tRNA synthetase are present as aminoacyl-tRNA synthetase sources. The amounts of synthesized peptide were evaluated with nonstop (upper right), stop (lower left), and stall (lower right). Also note that ICT1 functions more efficiently when the A-site is vacant (compare ICT1[WT] among nonstop, stop and sense). The results represent the average of at least three independent experiments. The bars on the graph indicate SD. For details, see the text. (**B**) ICT1 exhibits peptide-release activity on the polysome similar to that of puromycin. The polysome breakdown assay was performed as described previously [25]. *E. coli* polysomes (2.0 A_260_) were incubated with ribosome recycling factors (RRFmt, 15 µg; EF-G2mt, 30 µg) in the presence of the indicated peptide release factors (RF1Lmt or RF1mt, 60 µg; ICT1, 50 µg; puromycin, 10 µM), and subjected to sucrose gradient centrifugation.

In the next experiment we performed a multi-round translation assay. We utilized an *Escherichia coli*-based reconstituted *in vitro* system of coupled transcription-translation [11], which has the advantage that the 70S ribosomes do not contain integrated ICT1 (ArfB) factors. The peptide release factors were omitted from the system, in order to allow a specific analysis of the effects of ICT1 or other release factors. The ribosomes were programmed with mRNAs encoding a short polypeptide (MFFLF). We applied three different mRNAs: a nonstop mRNA without a 3′-end, and the stop (UAA) and stall (AGA) mRNAs, both of which have a 3′-UTR with 14 nucleotides (Fig. 2A, upper sketch in the upper panel, and Materials and Methods). The translation efficiencies were assessed by the incorporation of f[^14^C]Met. The ribosome recycling factor cannot hydrolyze a peptidyl-tRNA bound to ribosomes. Accordingly, the yield of the isolated polypeptides reflects the peptide-release activity of ICT1 with ribosomes stalled at the 3′ end of the mRNA or in the middle of the mRNA. ICT1 does not interfere with normal translation, because even excess of ICT1 in addition of the mito-release factor RF1Lmt does not affect the kinetics of the peptide release during the termination phase after the synthesis of the pentapeptide (Fig. S2B). Interestingly, ICT1 could release the oligo-peptide from stalled ribosomes programmed with the stop(UAA) or the stall(AGA) mRNAs, although to a lesser extent than with the nonstop(−) mRNA (Fig. 2A, lower panels). ICT1 functioned with the stop(UAA) and stall(AGA) mRNAs almost as efficiently as RF1Lmt did with the stop(UAA). After 30 minutes of synthesis, ICT1 yielded a release of ∼0.3 pmol polypeptides with the stop(UAA) and stall(AGA) mRNAs, and of ∼2.0 pmol with the nonstop(−) mRNA. RF1Lmt released ∼0.5 pmol polypeptides with the stop(UAA). These results suggest that ICT1 can rescue a stalled ribosome in the middle of the mRNA.

Next, we analysed, whether ICT1 can also release polypeptides from native polysomes, in which most of the ribosomes are in the middle of the mRNA. The control is a treatment with the antibiotic puromycin, which is an analogue of the 3′-end of an aminoacyl-tRNA, binds to the A-site region of the PTC, receives the polypeptide from the peptidyl-tRNA in the P site *via* a peptide-bond and leaves the ribosome as peptidyl-puromycin [12]. The action of puromycin on polysomes is indicated by a polysome-breakdown and an increase of the ribosomal subunits, (Fig. 2B, compare first two panels). The next three panels demonstrate that ICT1 is as effective as puromycin in breaking down the polysomes in contrast to RF1Lmt and RF1mt. ICT1 showed significant puromycin-like peptide-release activity on the polysomes, even though the A-sites on the polysomes are occupied with mRNA (the rightmost panel). RF1Lmt did not exhibit this activity, since polysomes do not carry a stop codon in their A-site (the third panel from the left).

Collectively, our results demonstrated that ICT1 can rescue stalled ribosomes in the middle of mRNA occupying the mRNA tunnel. These observations support the recent structural model of ICT1, according to which the C-terminal tail of ICT1 does not enter the mRNA path downstream of A-site of the ribosomal small subunit, at least as long as the ribosome stalling occurs in the middle of an mRNA [13]. Our findings also suggest that ICT1 is involved in the translation termination at non-standard stop codons AGA and AGG in mammalian mitochondria.

In all assays of both the multi-round translation and the polysome breakdown, RF1mt did not show peptide-release activity with any mRNA construct examined (Fig. 2A and B, RF1mt), consistent with the results of the peptide-release assay (Figs. 1B and C, RF1mt).

### The peptide-release activity of ICT1 requires the insertion sequence in the N-terminal globular domain

**Figure 3:**
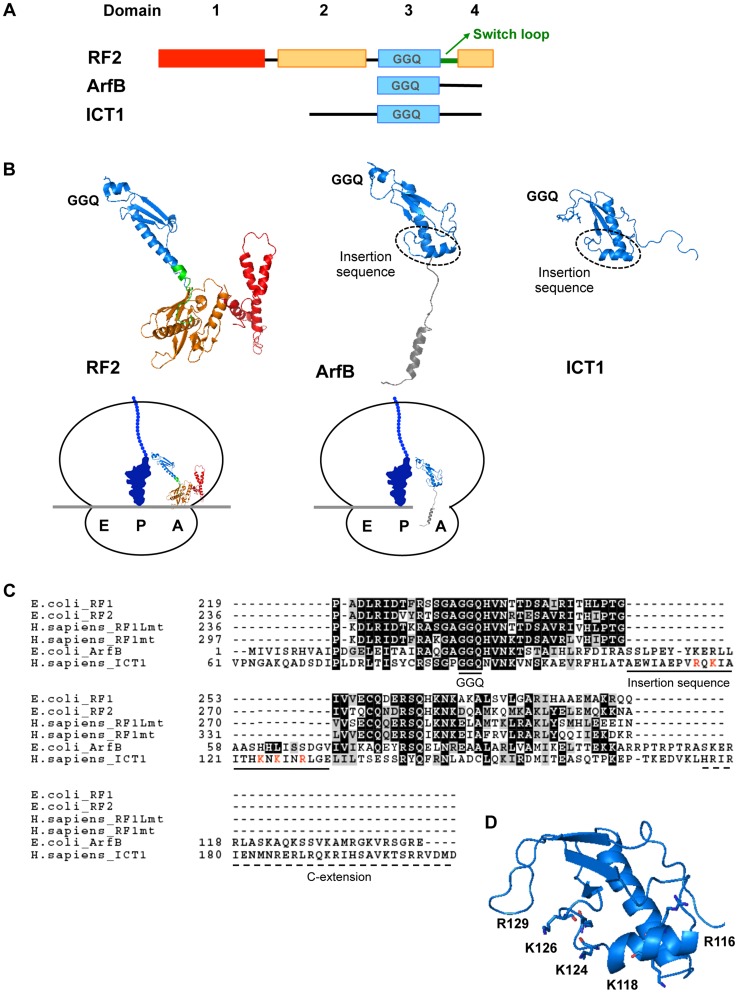
Structural comparison of ICT1 with the classical class 1 release factors. (**A**) Comparison of the domain-structures of *Thermus thermophilus* RF2, *E. coli* ArfB/YaeJ, and mouse ICT1. For *Thermus thermophilus* RF2, the catalytic domain 3 is indicated in blue, the stop codon recognition domain (consisting of domains 2 and 4) is shown in orange, and domain 1 is colored red. The switch loop is highlighted in green. For ArfB/YaeJ and ICT1, the N-terminal globular domain is shown in blue. (**B**) Comparison of 3D-structures of *Thermus thermophilus* RF2 [14] (PDB code 2WH1), *E. coli* ArfB/YaeJ [5] (PDB code 4DH9), and mouse ICT1 [26] (PDB code 1J26). Each domain of RF2, ArfB and ICT1 is shown in the same colors as in (A). The structure of the C-terminal tail of ICT1 has not been determined, and is not shown. The positions of the GGQ-loop and the insertion sequence are indicated. The sketches are the mirror pictures, so that the switch loop of RF2 or the insertion sequence of ICT1 is depicted in front, and the E-, P- and A-site are from left to right on the ribosome. (**C**) Amino acid sequence alignment of various class 1 release factors. Domain 3 of *E. coli* RF1, *E. coli* RF2, human RF1Lmt and human RF1mt were compared with *E. coli* ArfB (full length) and human ICT1 (amino acid residue position of 61th–206th), using the BoxShade program. The GGQ motif, the unique insertion sequence within ArfB and ICT1, and the C-terminal extension are underlined. The basic residues in the insertion sequence of ICT1 are highlighted in red. (**D**) The positions of the basic residues in the insertion sequence of ICT1. For the ICT1[*α2*] mutant, K124, K126 and R129 were substituted with alanine simultaneously. The sketch is the mirror picture, so that it is accorded with Fig. 3B.

The classical class 1 RF proteins need to recognize the stop codon and simultaneously to place their GGQ-motif in the peptidyl-transferase center (PTC) of the ribosome. Bacterial RF1/RF2 has the “switch loop” between domains 3 and 4 (Figs. 3A and B) [14], [15]. Stop codon recognition by domains 2 and 4 induces the rearrangement of this loop structure, thus positioning the GGQ-motif of domain 3 in the PTC (Fig. 3B, see the sketch of RF2 on the ribosome). On the other hand, the codon-independent peptide release factors, such as ArfB and ICT1, possess only domain 3 and lack the switch loop: domain 3 is connected by the flexible linker to its C-terminal tail that is thought to occupy the mRNA channel of the ribosome [5]. It is not clear how the GGQ-motif of ICT1 is optimally positioned in the PTC. In this regard, it is interesting that both ArfB and ICT1 uniquely contain an insertion sequence of 25 residues in the globular domain 3, in contrast to the classical class 1 RFs (Fig. 3B and C) [5], [7]. Therefore, we examined the function of the insertion sequence.

**Figure 4:**
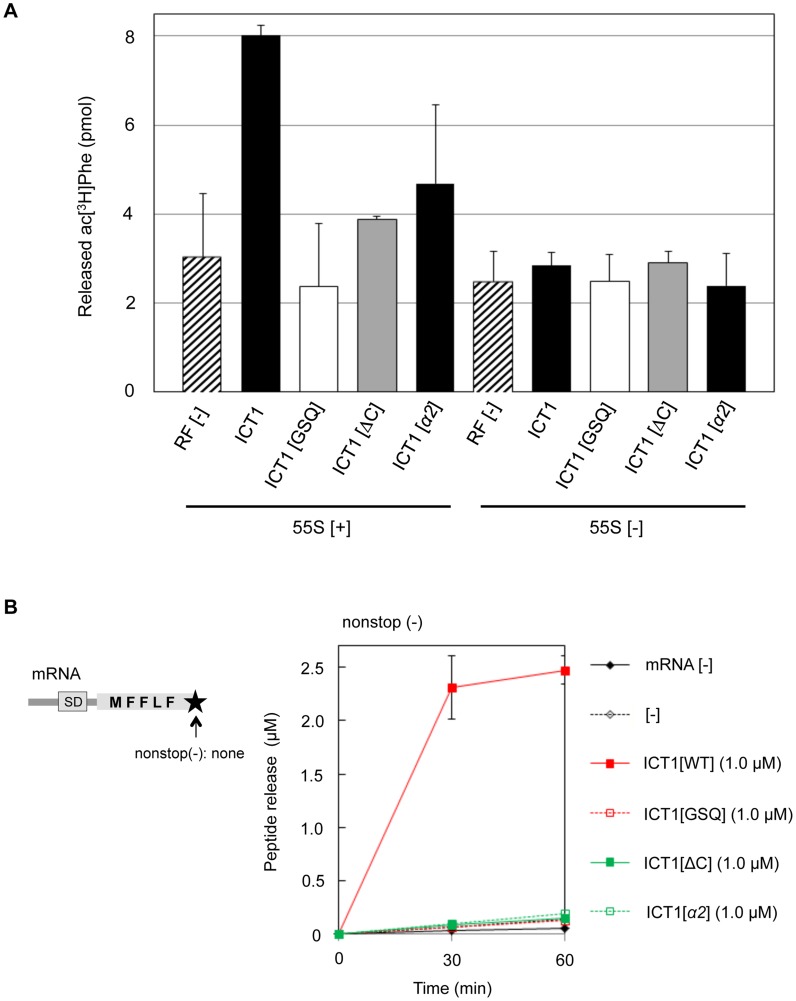
The peptide-release activity of ICT1 requires the insertion sequence in the N-terminal globular domain. (**A**) Peptide-release assays on 55S ribosomes without mRNA were performed as in Figure 1C, with the indicated peptide release factors. ICT1[GSQ], GGQ motif is mutated to GSQ; ICT1[ΔC], C-terminal 14 amino acid residues are truncated. 55S [+] and 55S [-] indicates the assays in the presence and absence of 55S mitoribosomes, respectively. Results represent the average of at least three independent experiments. The bars on the graph indicate SD. (**B**) Multi-round translation assays with *E. coli* 70S ribosomes were performed with the indicated peptide release factors, using the mRNAs depicted on the left. The utilized mRNA has no stop codon and no 3′UTR at the 3′-terminus. The amounts of synthesized peptide were evaluated. Red closed squares, ICT1[WT]; red open squares, ICT1[GSQ]; green closed squares, ICT1[ΔC]; green open squares, ICT1[*α2*]. Results represent the average of at least three independent experiments. The bars on the graph indicate SD. For details, see the text.

We first applied the peptide-release assay with 55S ribosomes in the absence of mRNA. There are five basic amino acid residues in the insertion sequence: R116, K118, K124, K126 and R129 (Fig. 3C, highlighted with red letters; and Fig. 3D). Assuming that the basic amino acid residues are involved in the interaction with ribosomal RNA, alanine substitutions were introduced at these positions. The peptide-release analyses revealed that the ICT1[*α2*] mutant, in which K124, K126 and R129 are simultaneously mutated to alanine, had little activity (Fig. 4A, ICT1[*α2*]). The activity of ICT1[*α2*] was almost as low as those of the ICT1[GSQ] and ICT1[ΔC] mutants, in which the GGQ-motif was changed to GSQ and the C-terminal 14 amino acids were truncated, respectively (Fig. 4A, ICT1[GSQ] and ICT1[ΔC]). The GGQ motif is critical for the peptide-release activity of ICT1 on the 70S ribosome [7]. The C-terminal 10 amino acids of ArfB, which correspond to the C-terminal 14 amino acids of ICT1, are essential for its ribosome binding capacity [4].

We also performed the multi-round translation assay to assess the peptide release activities of the ICT1 mutants. The ribosomes were programmed by a nonstop mRNA encoding a short polypeptide (MFFLF) without a stop codon (Fig. 4B, left). When wild type ICT1 was included in the system, the translation efficiency was increased, reflecting the enhanced multi-round translation (Fig. 4B, right, red closed squares). An alanine substitution of any one of the basic resides in the insertion sequence showed various defects (Fig. S4). However, when ICT1[*α2*] was included in the system, peptide release was abolished (Fig. 4B; green open squares). Neither ICT1[GSQ] nor ICT1[ΔC] enhanced the release (Fig. 4B; red open squares and green closed squares). These results suggest that the peptide-release activity of ICT1 is dependent on the insertion sequence in the N-terminal globular domain.

### The insertion sequence in the N-terminal globular domain of ICT1 is not involved in ribosome binding

**Figure 5:**
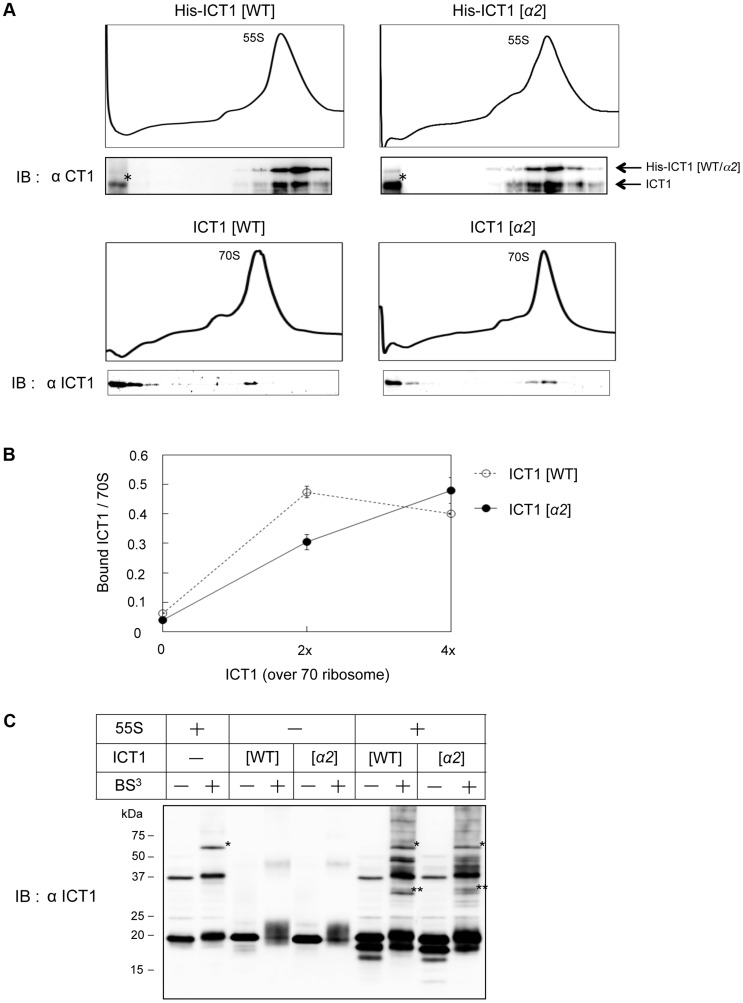
The insertion sequence in the N-terminal globular domain of ICT1 does not affect ribosome binding. (**A**) 55S mitoribosomes (upper panel) or *E. coli* 70S ribosomes (lower panel) were mixed with the indicated ICT1 proteins, and fractionated on 15%–30% (w/v) sucrose gradients. The fractions were analyzed by Western blotting with an ICT1 antibody. For the binding to 55S mitoribosomes, His-tagged ICT1 proteins were used. The arrows indicate the exogenous His-tagged ICT1 and endogenous ICT1. Intact His-tagged ICT1 proteins are hardly observed in the top fractions. Proteins indicated with asterisks are proteolytic products of His-tagged ICT1, rather than ICT1 that has been chased from 55S ribosomes. (**B**) The indicated amount of ICT1 proteins (open circles, ICT1[WT]; closed circles, ICT1[*α2*]) were incubated with *E. coli* 70S ribosomes (f.c. 0.25 µM). The ribosome-bound ICT1 was recovered by a filtering technique, and quantified by Western blotting against ICT1. The amount of ribosome-bound ICT1 per 70S ribosome was plotted against the amount of ICT1. Error bars represent SD of repeated measurements. (**C**) 55S ribosomes were mixed with the indicated ICT1 proteins and incubated in the presence of BS^3^ (f.c. 4 mM). The cross-linked products were analyzed by Western blotting with an ICT1 antibody. Note that the cross-linked proteins with the integral ICT1 (single asterisk) are different from those with the exogenous ICT1 (two asterisks, for example).

The mutations in the insertion sequence of ICT1 led to a loss of peptide-release activity (Fig. 4). Whether or not this loss was caused by a loss of binding to the ribosome was tested in the next experiment. Excess amounts of ICT1 proteins were incubated with 55S mitoribosomes or 70S *E. coli* ribosomes. The mixtures were then fractionated on a sucrose density gradient, and the ICT1 proteins were detected by immunoblotting of the fractions (Fig. 5A). For the analysis with 55S mitoribosomes, N-terminal his-tagged ICT1 proteins were used to discriminate the exogenous ICT1 from the endogenous ICT1; the exogenous his-tagged ICT1 could be separated from the endogenous ICT1 in SDS-PAGE, according to the increased molecular weight due to the his-tag. Both ICT1 and ICT1[*α2*] bound similarly to the 55S mitoribosomes (Fig. 5A, upper panels) and 70S ribosomes (Fig. 5A, lower panels). It is of note here that ICT1 proteins were incubated with 55S or 70S ribosomes in a 17-fold or 10-fold excess relative to the ribosomes, and approximately 5% and 10% of the input ICT1 was bound to the 55S and 70S ribosomes, respectively. This means that both 55S and 70S bound exogenous ICT1 with nearly a 1∶1 stoichiometry. These results clearly indicate that 55S ribosomes, already bearing the integrated ICT1, possess two ICT1 binding sites.

In Fig. 5B, the ribosome binding of wild type ICT1 or ICT1[*α2*] was analyzed more quantitatively. The 70S ribosomes were incubated with various amounts of ICT1 proteins, in up to 4-fold excess relative to 70S ribosomes. The ribosome-bound ICT1 proteins were then recovered by a filtering technique, and quantified by Western blotting against ICT1. The amounts of ribosome-bound ICT1 per 70S ribosome were plotted against the input-amounts of ICT1. The results confirmed that ICT1 and ICT1[*α2*] exhibit similar ribosome binding abilities.

The ribosome binding of wild type ICT1 or ICT1[*α2*] was further analyzed with 55S mitoribosomes in the presence of the crosslinking agent BS^3^, as shown in Fig. 5C. In this experiment, we aimed to obtain information about the binding site of exogenous ICT1 as well as that of the integrated ICT1 on the 55S ribosome. The 55S ribosomes were incubated with either wild type ICT1 or ICT1[*α2*] in the presence of BS^3^. The cross-linked products were fractionated by SDS-PAGE, and analyzed by Western blotting with an ICT1 antibody. Similar cross-linking products of wild type ICT1 and ICT1[*α2*] were observed (two asterisks), which was different when no ICT1 was added (one asterisk). These observations indicate that the binding sites of the 55S-integrated ICT1 and that of the exogenous ICT1 on the 55S ribosome are different.

Taken together, the results suggested that the insertion sequence of ICT1 does not affect the ribosome binding, which depends on the C-terminal tail of ICT1. We propose that the interaction of the insertion sequence with the ribosome is responsible for an optimal positioning of the GGQ-motif of ICT1 in the peptidyl-transferase center.

## Discussion

A previous study addressing the function of ICT1 used a heterologous combination of *E. coli* 70S ribosomes and human mitochondrial ICT1 [7]. In the present study, we employed homologous systems in most experiments: 55S mitochondrial ribosomes and purified mitochondrial factors including human mitochondrial ICT1. Our analyses revealed that exogenous ICT1 functions independently of the 55S-integrated ICT1; the 55S-integrated ICT1 shows no peptide-release activity. This is in agreement with a recent cryo-EM analysis at 4.9 Å resolution of the large subunit of porcine mitochondrial ribosomes, according to which ICT1 is located at the base of the central protuberance >70 Å away from the A site [16]. A conformational change of the large ribosomal subunit bringing the integrated ICT1 to the A-site is hardly conceivable.

**Figure 6:**
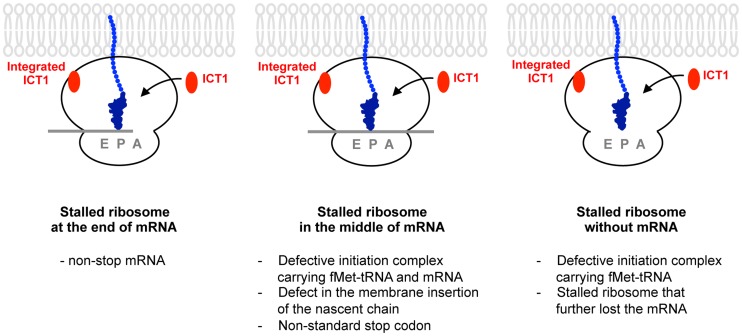
A model for the functions of ICT1 depicting the three scenarios involving the factor's activity. Ribosomes might stall for many reasons such as non-stop mRNA (left, Stalled ribosome at the end of mRNA), the defect in the membrane insertion of the nascent chain, the non-standard stop codon (middle, Stalled ribosome in the middle of mRNA), and so on. Stalled ribosome might further lose the mRNA (right, Stalled ribosome without mRNA). Defective initiation complex might exist (middle panel, stalled ribosome in the middle of mRNA; right, stalled ribosome without mRNA). ICT1 is a versatile rescue factor, which can take care of all type of stalled ribosomes by exerting its peptide-release activity at ribosomal A-site. Ribosome-free ICT1 is not yet detected in mitochondria. Accordingly, the ribosome-integrated ICT1, which is not present at A-site, is potentially released from the ribosomes in response to the ribosomal stall, and function at the ribosomal A-site. Alternatively, It is also possible that ICT1 is overexpressed in response to a ribosome stalling in order to produce ribosome-free ICT1, which acts on the stalled ribosome independently of the integrated ICT1.

So far, ribosome-free ICT1 has not been yet detected in mitochondria [7]. Thus, a possible explanation for our observation is that the ribosome-integrated ICT1 may be released from ribosomes, which are stalled under certain cellular conditions; the liberated ICT1 then binds to the ribosomal A-site to exert its peptide-release activity (Fig. 6). In mammalian mitochondria, all of the mtDNA-encoded proteins are membrane proteins, which are probably co-translationally inserted into the mitochondrial inner membrane [17]. Therefore, the substrates of ICT1 could be ribosomes that become stalled during the insertion of the nascent peptide into the membrane. Defects in polypeptide insertion into the membrane would cause the nascent polypeptide to become stuffed in the tunnel, and eventually stall the ribosome. It is possible that the stuffed nascent polypeptide in the tunnel of the stalled ribosome causes a structural change in the large ribosomal subunit, which might be important for recognition of ICT1 and might even elicit the release of ICT1 from the ribosome. Further studies, such as search for the ribosome-free ICT1, are required to support this hypothesis. It is also possible that ICT1 is overexpressed in response to a ribosome stalling in order to produce ribosome-free ICT1, which acts on the stalled ribosome independently of the integrated ICT1.

Our analyses revealed that ICT1 functions as a versatile rescue factor, *i.e.* it releases the polypeptide from the peptidyl-tRNA from ribosomes stalled at the end or in the middle of an mRNA or even from non-programmed ribosomes (Fig. 6). The ability of ICT1 to hydrolyze the peptidyl-tRNA on the non-programmed ribosome, *i.e.* in the absence of mRNA, may have some advantages in mitochondrial translation. For example, ICT1 may rescue a defective initiation complex, *e.g*. a ribosomal complex that binds fMet-tRNA in the absence of mRNA.

The ability of ICT1 to hydrolyze the peptidyl-tRNA on the ribosome in the middle of an mRNA would imply that ICT1 functions as a termination factor as well as a rescue factor. This assumption is supported by observations in *S. pombe*, according to which the growth defects observed in a Mrf1 (mitochondrial release factor)-deficient strain are compensated by overproduction of Pth4, which is a counterpart of human ICT1 and is also a component of the large subunit of the mitoribosome [18]. In human, mtDNA-encoded cytochrome c oxidase subunit I (CO1) and NADH dehydrogenase 6 (ND6) carry AGA and AGG codons at the end of their mRNAs, respectively. There are no tRNAs in mammalian mitochondria that decode AGA/G codons, *viz*. AGA/G codons are unassigned codons in mammalian mitochondria. Ribosomes stalled at these codons might be recognized by ICT1, *i.e.* it would function as a specific translation termination factor for CO1 and ND6 in human mitochondria. It has been shown that AGA/G codons in CO1 and ND6, presumably in association with other *cis* elements, promote −1 frameshifting in human mitoribosomes providing an UAG stop codon at the ribosomal A-site for both CO1 and ND6 mRNA. Accordingly, it was proposed that the translation of CO1 and ND6 are terminated by RF1Lmt [19]. However, a U preceding AGA/G is rare in mitochondria of most vertebrates [13]. Moreover, the peptidyl-tRNA at the P-site can no longer interact with the mRNA *via* codon-anticodon interactions after the frameshift, and thus the peptidyl-tRNA would probably not be able to keep the P/P state, which is a requirement for the peptide release by RF1Lmt. Therefore, it is still unclear whether RF1Lmt or ICT1 catalyze the peptide release of CO1 and ND6.

The mutations of basic residues in the insertion sequence of ICT1 caused a loss of peptide release activity, but did not affect its ribosome binding ability (Figs. 4 and 5, respectively). The crystal structure of ArfB/YaeJ on the 70S ribosome revealed that the insertion sequence in the N-terminal globular domain interacts with 23S rRNA [5]. The phosphate backbones of U1946 and C1947 in 23S rRNA form hydrogen-bonds with the main chain N atom of His62 and the Oγ atom of Ser60 of ArfB, respectively (Fig. S5). Although these residues are not conserved in ICT1, it is plausible that other residues in the insertion region interact with rRNA in the large mitochondrial ribosomal subunit. The mutations of basic residues in the insertion region of ICT1 might disrupt the proper interaction between the insertion sequence and rRNA, without affecting the ribosome binding ability of ICT1, which is governed by the C-terminal tail of ICT1. The role of the insertion sequence of ICT1 or ArfB, the codon-independent peptide release factors, could be to place the GGQ-motif in the appropriate orientation for peptide release. Very recently, effects of the point mutations in the insertion sequence of ArfB have been studied [20]. Decreased peptide release activities were observed for some mutants, probably due to the inappropriate orientation of the GGQ-motif. In bacteria, ArfA assists RF2 for the codon-independent peptide-release activity [2], [3]. Although a structural study is required to verify how ArfA functions with RF2, it is possible that ArfA plays the same role as the insertion sequence of ICT1/ArfB.

ICT1 is one of four proteins of the class 1 RF-family in mammalian mitochondria: RF1Lmt/mtRF1a, RF1mt, C12orf65 and ICT1 [21]. Despite great efforts, the roles of RF1mt and C12orf65 are not known. A previous structural simulation study proposed that the RF1mt targets the ribosomal complex with a vacant A-site, and thus is a candidate for the ribosome rescue factor in mammalian mitochondria [21]. Another simulation study proposed that the RF1mt would display peptide release activity for UAA and UAG stop codons, but exclusively on 55S mitoribosome [13]. However, using such ribosomal complexes, we could not detect any peptide release activity of RF1mt by itself (Figs. 1B, 1C and 3B). It is possible that RF1mt shows peptide release activity through its GGQ motif in collaboration with unknown factors, as bacterial RF2 shows codon-independent peptide release activity in concert with ArfA [2], [3].

In spite of some answers concerning translation termination in mitochondria given in this study, still important questions remain, *e.g.* under which conditions ribosome-free ICT1 is produced to act on the ribosomal A-site.

## Materials and Methods

### Preparation of 55S ribosomes

55S ribosomes were prepared from pig liver mitochondria, as described previously [22]. The ribosome concentrations were calculated assuming 32 pmol/A_260_ for 55S ribosomes.

### Expression and purification of Human ICT1

HMICT1/pET15b was used as the *E. coli* expression vector for the N-terminally histidine-tagged ICT1. The EST clone (IOH11951) was purchased from Invitrogen. The coding sequence was amplified by PCR, using the primers 5′-CGGTGCCCACGCCATATGCTGCACAAGCAGAAAGACGGCACTG-3′ and 5′-GAGGCTCGAGTCAGTCCATGTCGACCCTCCTGCTT-3′. The obtained coding sequences were cloned between the *Nde*I and *Xho*I sites of a modified pET15b vector (Novagen), in which the original multi-cloning sites were modified in our laboratory.

HMICT1/pET15b was transformed into *E. coli* Rosetta(DE3)/pLysS. Cultures were induced with 100 µM isopropyl-1-thio-D-galactopyranoside at 18°C overnight. The protein was purified by Ni-NTA (QIAGEN) column chromatography. After histidine tag digestion using thrombin protease (GE Healthcare), the protein was further purified by Q-Sepharose column chromatography (GE Healthcare). The protein was concentrated to 5 mg/ml, divided into aliquots, flash-frozen, and stored at −80°C.

The *E. coli* expression vectors for the ICT1 mutants were prepared by PCR, using HMICT1/pET15b. The primers for PCR were as follows: 5′-GTGGTCCTGGGTCGCAGAATGTGAAC-3′ and 5′-GTTCACATTCTGCGACCCAGGACCAC-3′ for GSQ, 5′-TGACTCGAGGGTACCCCGCGGGCGG-3′ and 5′-TCTCTTTTGTCTCAGCCTTTCCCGATTC-3′ for ΔC, 5′-ATCGCGGAGCCCGTGGCGCAGAAGATAGCCAT-3′ and 5′-ATGGCTATCTTCTGCGCCACGGGCTCCGCGAT-3′ for R116A, 5′-GAGCCCGTGCGGCAGGCGATAGCCATCACGCA-3′ and 5′-TGCGTGATGGCTATCGCCTGCCGCACGGGCTC-3′ for K118A, 5′-ATAGCCATCACGCATGCAAACAAGATCAACAG-3′ and 5′-CTGTTGATCTTGTTTGCATGCGTGATGGCTAT-3′ for K124A, 5′-ATCACGCATAAAAACGCGATCAACAGGTTAGG-3′ and 5′-CCTAACCTGTTGATCGCGTTTTTATGCGTGAT-3′ for K126A, 5′-AAAAACAAGATCAACGCGTTAGGAGAGTTGAT-3′ and 5′-ATCAACTCTCCTAACGCGTTGATCTTGTTTTT-3′ for R129A, and 5′-ATAGCCATCACGCATGCAAACGCGATCAACGCGTTAGGAGAGTTGATCC-3′ and 5′-GGATCAACTCTCCTAACGCGTTGATCGCGTTTGCATGCGTGATGGCTAT-3′ for *α2*(124-126-129A). These mutants were expressed and purified as described above.

### *In vitro* 55S peptide-release assay

The heteropolymeric MFV-mRNA and the MF-stop mRNA 5′-GGGAAAAGAAAAGAAAAGAAA-**AUG-UUC-GUU/UAA**-AAAAGAAAAGAAAAGAAAAUAUUGAAUU-3′, containing three codons (Met-Phe-Val or Met-Phe-stop, indicated with bold letters in mRNA sequence) in the middle, respectively, were prepared by run-off transcription, as reported [23]. *E. coli* tRNA^Phe^ was purchased from Sigma-Aldrich. The ac[^3^H]Phe-tRNA^Phe^ (N-acetyl-[^3^H]Phe-tRNA^Phe^) was purified by reverse-phase HPLC on a Nucleosil 300-5 C4 column using a methanol gradient, as described previously [24]. Human RF1Lmt and RF1mt were expressed in *E. coli* and purified as described previously [9].

All complexes were prepared in a buffer containing 20 mM HEPES-KOH (pH 7.6), 4.5 mM Mg(OAc)_2_, 150 mM KOAc, 0.05 mM spermine, 2.0 mM spermidine, and 4 mM 2-mercaptoethanol. Ternary complexes were formed in a reaction volume of 50 µl, containing 20 pmol 55S ribosomes, 200 pmol MFV/MFstop mRNA and 20 pmol ac[^3^H]Phe-tRNA^Phe^. The reaction mix was incubated for 15 min at 37°C. A binding assay confirmed that approximately 0.9 pmol of ac[^3^H]Phe-tRNA^Phe^ was bound to the ribosomes, at this point. The reactions were further incubated (75 µl total volume) for 45 min at 25°C with 80 pmol of ICT1, RF1Lmt or RF1mt. To stop the reaction, an equal volume of 1 N HCl was added to the reaction. The released ac[^3^H]Phe was extracted by ethyl acetate, and the amount of [^3^H]Phe incorporated into polypeptides was determined using a scintillation counter. The results were evaluated relative to the 100% value when all of the ac[^3^H]Phe-tRNA^Phe^ was bound to the ribosome; *i.e.*, 0.9 pmol was hydrolyzed. The reactions in the absence of mRNA were also performed as above, with the initial incubation containing 55S ribosomes and ac[^3^H]Phe-tRNA^Phe^, except for the second incubation for 30 min at 25°C, where 16 pmol of ICT1, RF1Lmt or RF1mt were added.

Fig. 1B confirmed that acPhe-tRNA is programmed to P-site, and the stop codon is properly positioned to A-site on the ribosome, since RF1Lmt showed peptide-release activity with MF-stop(UAA) mRNA, but not with MFV mRNA.

When the puromycin assay was performed using the ribosomal complex prepared in Fig. 1B, approximately 9.6 pmol of acPhe (>650%) was transferred to puromycin, while the binding assay confirmed that only 0.9 pmol of acPhe-tRNA (100%) is bound to ribosome in the presence of mRNA. This indicates that acPhe-tRNA goes to P-site on the mRNA-containing ribosome as well as on the mRNA-free ribosome. Note that acPhe-tRNA on the latter ribosome can react with puromycin but not with RFs (RF binds to ribosome in a stop-codon dependent manner.).

### Polysome breakdown assay

Polysomes were prepared from the *E. coli* A19 strain, according to the procedures described previously [25]. The standard reaction mixtures (250 µl) contained *E. coli* polysomes (2.0 A_260_), RRFmt (15 µg), EF-G2mt (30 µg) and peptide release factors (RF1Lmt or RF1mt, 60 µg; ICT1, 50 µg; 10 µM puromycin). The mixtures were incubated at 30°C for 20 min in buffer (10 mM Tris-HCl [pH 7.5], 80 mM NH_4_Cl, 8.2 mM MgSO_4_, 1 mM DTT, and 0.5 mM GTP) and fractionated on 15%–30% (w/v) sucrose gradients (containing 10 mM Tris-HCl [pH 7.4], 80 mM NH_4_Cl, 8.2 mM MgSO_4_, and 1 mM DTT) by centrifugation at 39,000 rpm for 2 hour, using an SW41Ti rotor (Beckman Coulter). The gradients were recovered from top to bottom, using a density gradient fractionator (Towa Labo, Model 152–001) while monitoring the absorbance at 260 nm.

### *In vitro* multi-round translation assay

DNA templates were prepared as follows. Using the plasmid pURE1 (Post Genome Institute Co., Ltd.), DNA fragments were amplified by PCR with the T7 promoter primer 5′-GCGCGTAATACGACTCACTATAG-3′ and 3′ primer 5′-GATCCCTAGAACAGTTAGAACAGGAAGAACATATGATATCTCCTTCTTAAAGTT-3′ (stop), 5′-GATCCCTAGAACAGTCTGAACAGGAAGAACATATGATATCTCCTTCTTAAAGTT-3′ (stall) and 3′ primer 5′-GAACAGGAAGAACATATGATATCTCCTTCTTAAAGTT-3′ (nonstop). The corresponding mRNAs used in Fig. 2 have the following sequences. Nonstop mRNA: 5′-GGGAGACCACAACGGUUUCCCUCUAGAAAUAAUUUUGUUUAACUUUAAGAAGGAGAUAUCAUAUGUUCUUCCUGUUC-3′ ; stop (UAA): 5′-GGGAGACCACAACGGUUUCCCUCUAGAAAUAAUUUUGUUUAACUUUAAGAAGGAGAUAUCAUAUGUUCUUCCUGUUCUAACUGUUCUAGGGAUC-3′ ; and stall (AGA): 5′-GGGAGACCACAACGGUUUCCCUCUAGAAAUAAUUUUGUUUAACUUUAAGAAGGAGAUAUCAUAUGUUCUUCCUGUUCAGACUGUUCUAGGGAUC-3′. (the underlined sequence encodes the MFFLF short polypeptide). [^14^C]Methionine-labeled fMet-tRNA was prepared as described previously [25].

The system was reconstituted as reported previously [11], [25], with slight modifications. Briefly, RRF was omitted from the standard system, and EF-G2mt (0.8 µg) and RRFmt (0.2 µg) were included as the recycling factors. *E. coli* RFs were omitted, and RF1Lmt (0.5 µg), RF1mt (2 µg) and ICT1 (1.1 µg) were included. Only phenylalanyl-tRNA synthetase and leucyl-tRNA synthetase were included as aminoacyl-tRNA synthetase sources. Instead of amino acid mixtures, phenylalanine and leucine were used, and [^35^S]methionine was omitted from the system. The other components were the same as those described previously.

The reactions (50 µL) were started by the addition of 0.2 pmol of PCR-amplified DNA template and 100 pmol of f[^14^C]Met-tRNA. After an incubation at 37°C for the indicated time periods, aliquots were withdrawn and added to an equal volume of 1 N HCl, to stop the reaction. The translated polypeptides were extracted with ethyl acetate, and the incorporation of [^14^C] methionine into polypeptides was determined using a scintillation counter.

### Ribosome/ICT1 binding assay

For the SDG analysis with *E. coli* 70S ribosomes, reaction mixtures (250 µl) containing 25 pmol 70S ribosomes and 250 pmol ICT1, in buffer A (10 mM Tris-HCl [pH 7.5], 80 mM NH_4_Cl, 8.2 mM MgSO_4_ and 1 mM DTT), were incubated at 30°C for 20 min and fractionated on 15%–30% (w/v) sucrose gradients, in the same manner as the polysome breakdown assay. In the analysis with 55S mitoribosomes, reaction mixtures (250 µl) containing 75 pmol 55S ribosomes and 1,250 pmol N-terminal histidine tagged ICT1 were incubated as in the assay with 70S ribosomes and fractionated on 15%–30% (w/v) sucrose gradients by centrifugation at 39,000 rpm for 5.5 hour, using an SW41Ti rotor (Beckman Coulter). The fractions were analyzed by Western blotting.

For the dot-blot analysis, reaction mixtures (50 µl) containing 12.5 pmol *E. coli* ribosome and the indicated amount of ICT1 in buffer A were incubated at 30°C for 20 min, loaded on a Microcon-YM100 column (Millipore), and centrifuged at 3,000× g at 4°C for 5 minutes. The columns were washed twice with 100 µl of buffer A. For sample recovery, the columns were incubated with 50 µl buffer A at room temperature for 1 minute. The samples were collected in fresh tubes from the inverted columns by centrifugation at 10,000× g at 4°C for 5 minutes, with rinses of 100 µl of buffer A. After the recovered samples were brought up to a volume of 500 µl with buffer A, 50 µl aliquots containing EDTA (f.c. 20 mM) were used as the dot-blot loading samples. For quantification, purified recombinant ICT1 was used as loading samples for a standard curve.

### Crosslinking assay

55S ribosomes (46 pmol in 100 µl final volume) were mixed with a 5-fold excess of ICT1, in buffer containing 20 mM Hepes-KOH (pH 7.6), 8.2 mM MgSO_4_, and 80 mM NH_4_Cl. The mixtures were incubated at 30°C for 20 min. The reactions were supplemented with the amino-reactive crosslinking agent BS^3^ (Bis[sulfosuccinimidyl]suberate, Thermo Scientific) to a final concentration of 4 mM, and were incubated at 25°C for 30 min. BS^3^ crosslinking was stopped by the addition of SDS-PAGE loading dye. The samples were analyzed by Western blotting.

### Western blotting analysis

The polyclonal antisera for ICT1 were generated in our laboratory, by injecting rabbits with the purified recombinant proteins.

## Supporting Information

Figure S1 RF1mt shows no peptide release activity on 55S ribosomes, even using an excess amount. (**A**) The reaction mixture (50 µl), containing 20 pmol 55S mitoribosomes, 200 pmol MFV/MFstop mRNA and 20 pmol ac[^3^H]Phe-tRNA^Phe^, was incubated for 15 min at 37°C. The reactions were further incubated (75 µl total volume) for 45 min at 25°C with indicated amounts of RF1mt or RF1Lmt. ×4 RF1mt/RF1Lmt corresponds to 80 pmol proteins, which is 4-fold excess relative to the 55S mitoribosomes. The released ac[^3^H]Phe was extracted by ethyl acetate, and the amount of ac[^3^H]Phe was determined by scintillation counting. (**B**) The reactions in the absence of mRNA were also performed as above, the initial incubation contained the 55S ribosomes and ac[^3^H]Phe-tRNA^Phe^.
(PDF)

Figure S2 ICT1 does not interfere with normal translation. (**A**) ICT1 shows aminoacyl-tRNA hydrolase activity on 55S mitoribosomes in the absence of mRNA. The assay was performed as in Fig. 1C, using [^3^H]Phe-tRNA^Phe^ instead of ac[^3^H]Phe-tRNA^Phe^. The reaction mixture (50 µl), containing 20 pmol [^3^H]Phe-tRNA^Phe^ and 20 pmol 55S mitoribosomes, was incubated for 15 min at 37°C. The reactions were further incubated (75 µl total volume) for 30 min at 25°C with 16 pmol of RF1Lmt, RF1mt or ICT1. The reactions were terminated with 500 µl 5% TCA and centrifuged at 10,000× g at 4°C for 15 minutes. The supernatants were collected and the amount of released [^3^H]Phe were determined with scintillation counter. 55S [+] and 55S [−] indicates the assays in the presence and absence of 55S mitoribosomes, respectively. Note that TCA precipitation method was applied to recover released [^3^H]Phe, due to the inefficient extraction of [^3^H]Phe by ethyl acetate. Since Phe-tRNA is more unstable than acPhe-tRNA, and is easily deacylated during incubation, the background value of the assay (∼11.0 pmol) is higher than that of Fig. 1C (∼3.0 pmol). (**B**) *In vitro* multi-round assays of coupled transcription-translation were performed using the “stop” mRNA, in the presence of the indicated peptide release factors. For details see Figure 2A and the text. ICT1 [WT] or ICT1 [GSQ] was competed with RF1Lmt. ICT1 does not inhibit either the peptide release reaction by RF1Lmt or the peptide elongation reaction.
(PDF)

Figure S3 ICT1 as well as ArfB show peptide release activity on 70S ribosomes in the absence of mRNA. The reaction mixture (50 µl), containing 20 pmol ac[^3^H]Phe-tRNA^Phe^ and 20 pmol *E. coli* 70S ribosomes, was incubated for 15 min at 37°C. The reactions were further incubated (75 µl total volume) for 45 min at 25°C with 80 pmol of RF1Lmt, RF1mt, ICT1, ICT1 mutants or ArfB. The released ac[^3^H]Phe was extracted by ethyl acetate, and the amount of ac[^3^H]Phe was determined with a scintillation counter.
(PDF)

Figure S4 Single alanine substitution mutations in the insertion sequence of ICT1 have minimal effects on the peptide release activity of ICT1. *In vitro* multi-round translation assays were performed with the indicated ICT1 mutants, using the “stall” mRNA. Details about the mutants in Figure 3D; the procedure is illustrated in Figure 2A.
(PDF)

Figure S5 Interaction of the insertion sequence of ICT1 with 23S rRNA. Left, overview of the structure of ICT1 on the 70S ribosome [5]. ICT1, red; tRNA^fMet^, blue; 50S, green; 30S, light blue. The insertion sequence in the N-terminal domain of ICT1 is colored orange. Right, close-up view of the interaction between the insertion sequence of ICT1 and 23S rRNA. For details see Discussion.
(PDF)
