# Supplementary figures and images for: Ribosome Rescue and Translation Termination at Non-Standard Stop Codons by ICT1 in Mammalian Mitochondria
Source: PLoS Genet. 2014 Sep 18;10(9):e1004616. doi: 10.1371/journal.pgen.1004616 (PMC4169044; doi:10.1371/journal.pgen.1004616)

**A**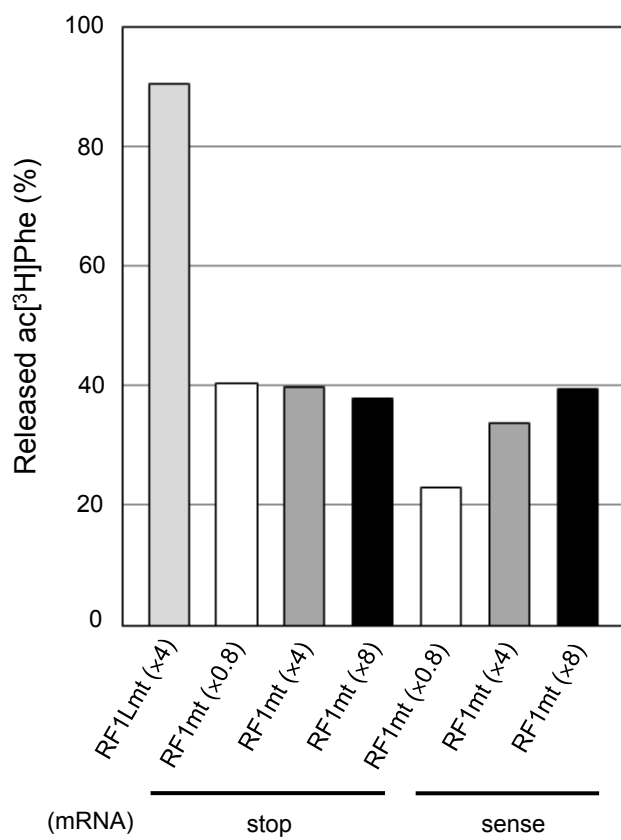**B**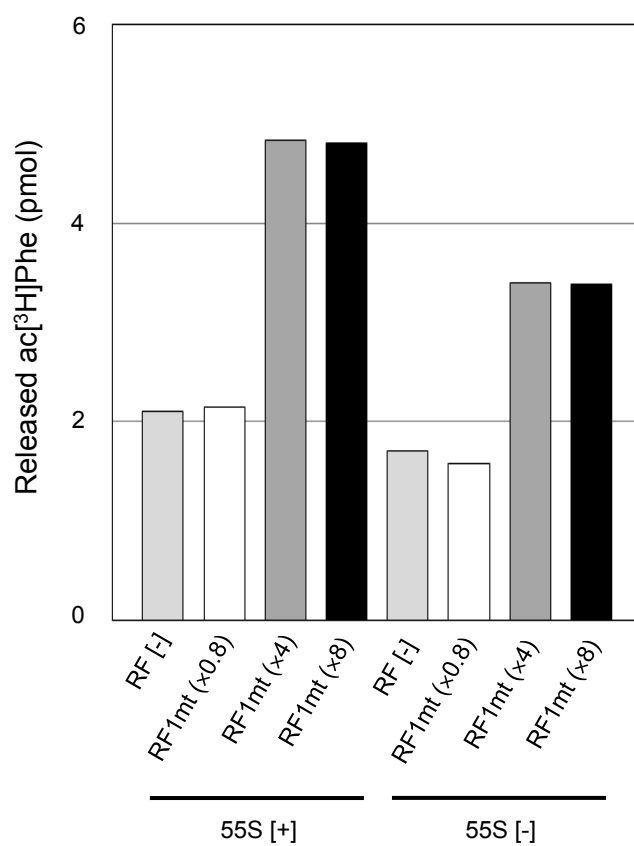

Supplement: Figure S1 — RF1mt shows no peptide release activity on 55S ribosomes, even using an excess amount. (A) The reaction mixture (50 µl), containing 20 pmol 55S mitoribosomes, 200 pmol MFV/MFstop mRNA and 20 pmol ac[3H]Phe-tRNAPhe, was incubated for 15 min at 37°C. The reactions were further incubated (75 µl total volume) for 45 min at 25°C with indicated amounts of RF1mt or RF1Lmt. ×4 RF1mt/RF1Lmt corresponds to 80 pmol proteins, which is 4-fold excess relative to the 55S mitoribosomes. The released ac[3H]Phe was extracted by ethyl acetate, and the amount of ac[3H]Phe was determined by scintillation counting. (B) The reactions in the absence of mRNA were also performed as above, the initial incubation contained the 55S ribosomes and ac[3H]Phe-tRNAPhe. (PDF) [file pgen.1004616.s001.pdf]

**A**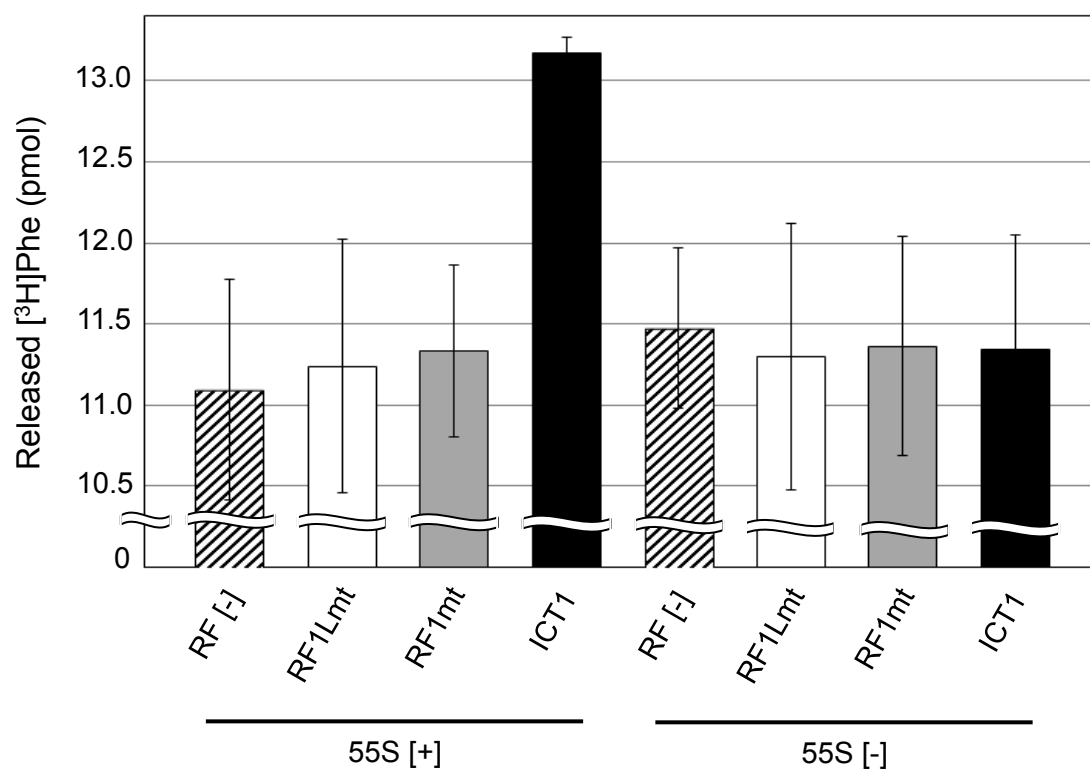**B**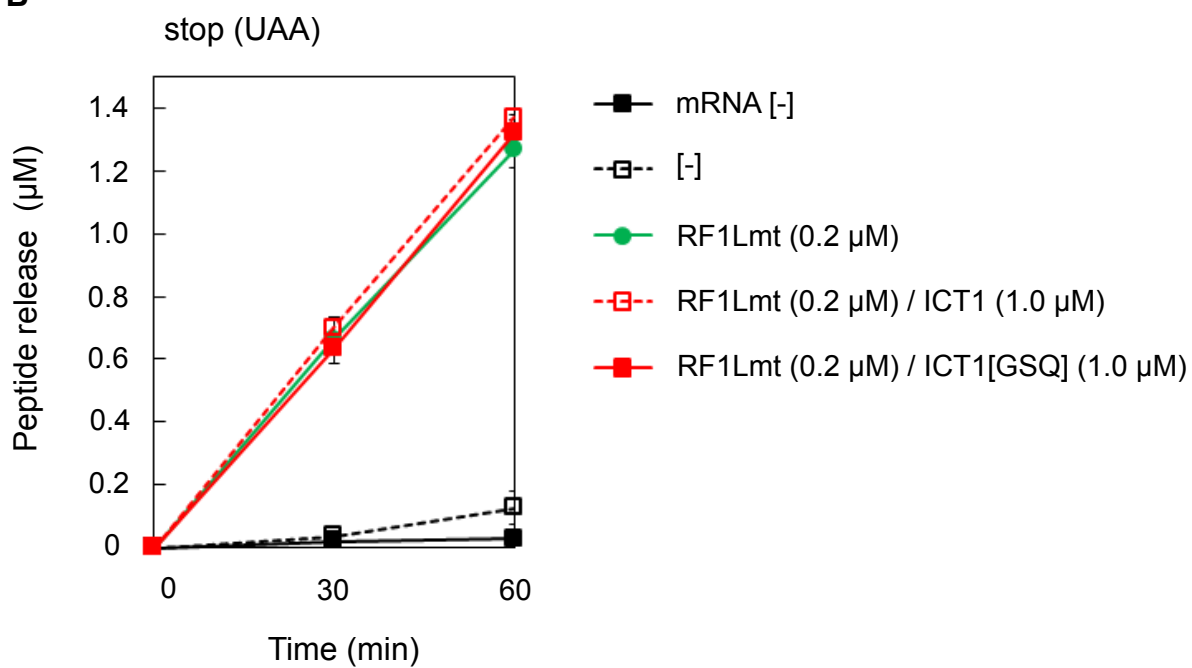

Supplement: Figure S2 — ICT1 does not interfere with normal translation. (A) ICT1 shows aminoacyl-tRNA hydrolase activity on 55S mitoribosomes in the absence of mRNA. The assay was performed as in Fig. 1C, using [3H]Phe-tRNAPhe instead of ac[3H]Phe-tRNAPhe. The reaction mixture (50 µl), containing 20 pmol [3H]Phe-tRNAPhe and 20 pmol 55S mitoribosomes, was incubated for 15 min at 37°C. The reactions were further incubated (75 µl total volume) for 30 min at 25°C with 16 pmol of RF1Lmt, RF1mt or ICT1. The reactions were terminated with 500 µl 5% TCA and centrifuged at 10,000× g at 4°C for 15 minutes. The supernatants were collected and the amount of released [3H]Phe were determined with scintillation counter. 55S [+] and 55S [−] indicates the assays in the presence and absence of 55S mitoribosomes, respectively. Note that TCA precipitation method was applied to recover released [3H]Phe, due to the inefficient extraction of [3H]Phe by ethyl acetate. Since Phe-tRNA is more unstable than acPhe-tRNA, and is easily deacylated during incubation, the background value of the assay (∼11.0 pmol) is higher than that of Fig. 1C (∼3.0 pmol). (B) In vitro multi-round assays of coupled transcription-translation were performed using the “stop” mRNA, in the presence of the indicated peptide release factors. For details see Figure 2A and the text. ICT1 [WT] or ICT1 [GSQ] was competed with RF1Lmt. ICT1 does not inhibit either the peptide release reaction by RF1Lmt or the peptide elongation reaction. (PDF) [file pgen.1004616.s002.pdf]

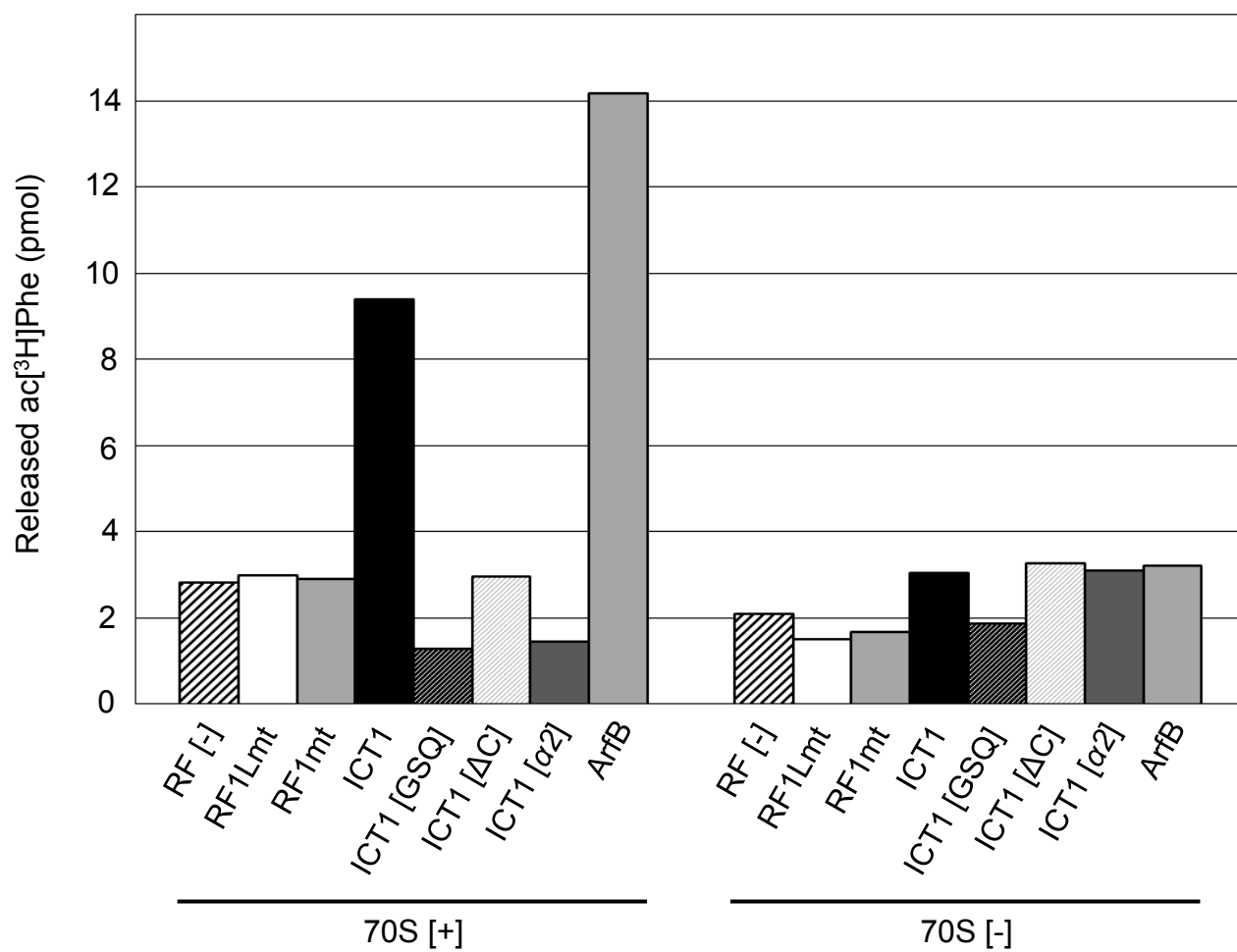

Supplement: Figure S3 — ICT1 as well as ArfB show peptide release activity on 70S ribosomes in the absence of mRNA. The reaction mixture (50 µl), containing 20 pmol ac[3H]Phe-tRNAPhe and 20 pmol E. coli 70S ribosomes, was incubated for 15 min at 37°C. The reactions were further incubated (75 µl total volume) for 45 min at 25°C with 80 pmol of RF1Lmt, RF1mt, ICT1, ICT1 mutants or ArfB. The released ac[3H]Phe was extracted by ethyl acetate, and the amount of ac[3H]Phe was determined with a scintillation counter. (PDF) [file pgen.1004616.s003.pdf]

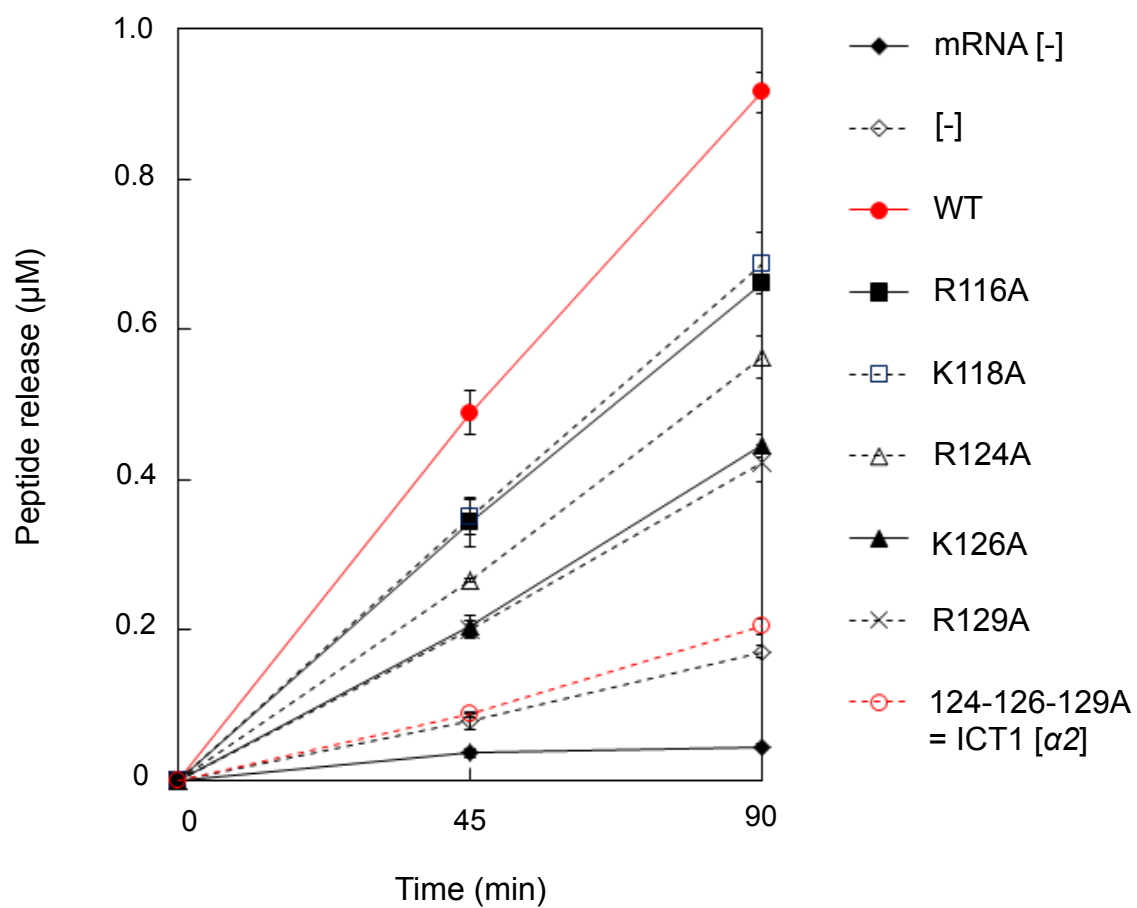

Supplement: Figure S4 — Single alanine substitution mutations in the insertion sequence of ICT1 have minimal effects on the peptide release activity of ICT1. In vitro multi-round translation assays were performed with the indicated ICT1 mutants, using the “stall” mRNA. Details about the mutants in Figure 3D; the procedure is illustrated in Figure 2A. (PDF) [file pgen.1004616.s004.pdf]

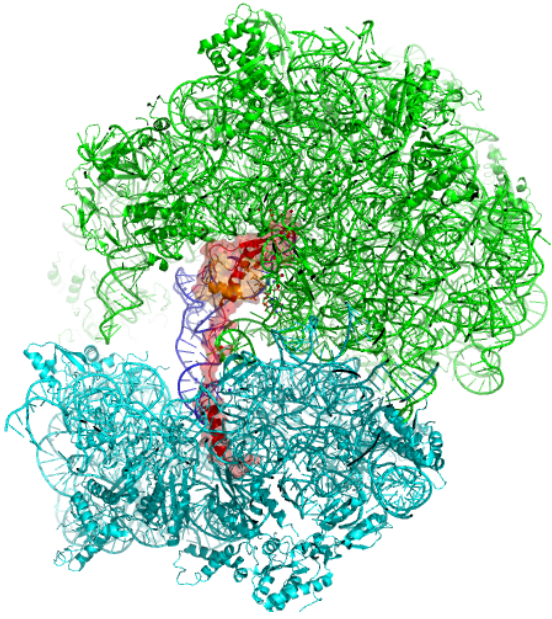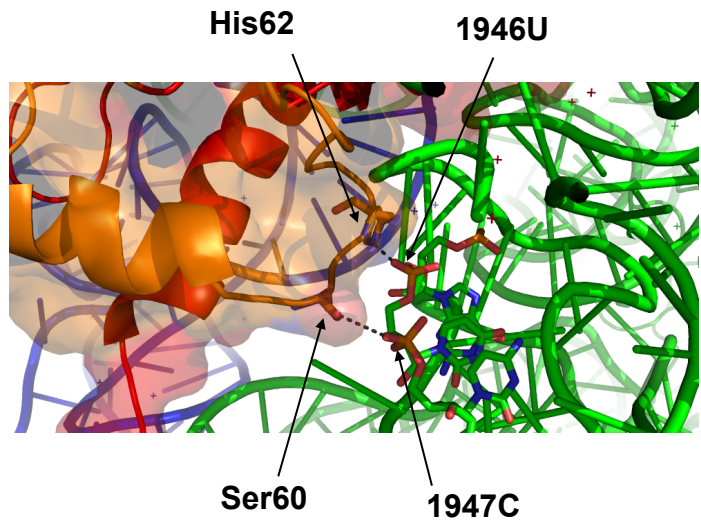

Supplement: Figure S5 — Interaction of the insertion sequence of ICT1 with 23S rRNA. Left, overview of the structure of ICT1 on the 70S ribosome [5]. ICT1, red; tRNAfMet, blue; 50S, green; 30S, light blue. The insertion sequence in the N-terminal domain of ICT1 is colored orange. Right, close-up view of the interaction between the insertion sequence of ICT1 and 23S rRNA. For details see Discussion. (PDF) [file pgen.1004616.s005.pdf]
